# Defect states and kinetic parameter analysis of ZnAl_2_O_4_ nanocrystals by X-ray photoelectron spectroscopy and thermoluminescence

**DOI:** 10.1038/s41598-019-57227-8

**Published:** 2020-01-15

**Authors:** Megha Jain, Manju, Ravi Kumar, Sung Ok Won, Keun Hwa Chae, Ankush Vij, Anup Thakur

**Affiliations:** 1 Punjabi University, Advanced Materials Research Lab, Department of Basic and Applied Sciences, Patiala, 147 002 India; 2 Punjabi University, Department of Physics, Patiala, 147 002 India; 3 National Institute of Technology, Center for Material Science & Engineering, Hamirpur, 177 005 India; 4 Korea Institute of Science and Technology, Advanced Analysis Center, Seoul, 02792 South Korea; 5 Amity University Haryana, Nanophosphors Lab, Department of Physics, Gurgaon, 122 413 India

**Keywords:** Materials science, Nanoscience and technology

## Abstract

Defect states in ZnAl_2_O_4_ have a significant role in its applicability as a luminescent material. To understand the nature and distribution of defects in its crystal lattice, thermoluminescence (TL) study has been carried out. Excellent TL response is observed from *γ*- and ultraviolet-irradiated samples at different doses and exposure durations, respectively. Different type of fuels employed in combustion synthesis show a remarkable effect on the trap distribution and hence luminescence properties. Shallow and deep traps are observed in crystals attributed to O^−^ vacancies and F^+^ centers. The mechanism of trapping, retrapping and recombination have been depicted through schematic band model diagram. X-ray photoelectron spectroscopy indicated the presence of various types of defects specifically Al_*Zn*_ antisite defect, oxygen and zinc vacancies which are further upheld by photoluminescence and Raman spectroscopy. All results when summed up, predict ZnAl_2_O_4_ to be a quality material for dosimetry.

## Introduction

In the search of better and efficient luminescent material for commercial applications, scientific community has been continuously exploring photoluminescence (PL) and thermoluminescence (TL) in different materials^1–4^. Metal oxides are drawing considerable attention owing to their non-toxic nature and tunable luminescence properties attributable to the defect states. Zinc aluminate (ZnAl_2_O_4_) is a well known member of spinel family possessing versatile properties and magnificent applications^5–10^. It crystallizes in *fcc* crystal system with *Fd*$\bar{3}$*m* space group. It is a wide band gap material with band gap value of ~3.8 eV in bulk form^11^. It is a *n*-type semiconducting material, its conductivity prominently attributed to Al_*Zn*_ antisite defects which is a shallow defect near conduction band edge accompanied by cationic vacancies^12^. Due to complex crystal structure and multiple defect centers such as cationic-anionic vacancies, antisite defects and cationic interstitial, it shows alluring luminescence^6–9^. Most of the work has been reported on photoluminescence of undoped as well as doped ZnAl_2_O_4_ ^8–10,12–16^. However, in order to understand the defect levels and their nature in a material, it must be studied rigorously, which can be brought about by TL study.

TL studies can help in locating the trap states and their nature in semiconducting crystal systems^17–20^. TL is the heat stimulated emission from a material, being exposed to an ionizing radiation prior to thermal treatment^21^. Various types of ionizing radiations such as *α*, *β*, *γ*, cosmic, ultraviolet (UV) and X-rays can be utilized for the pre-exposure^22,23^. Informative parameters such as order of kinetics, trap depth and frequency factor from TL emission must be known so that basic mechanism of TL emission and dosimetric properties of material could be critically analyzed^24^. Order of kinetics enables one to understand the type of trapping and recombination process. First order kinetics indicates a faster decay while second order kinetics indicates retrapping of charge carriers leading to delayed emission^25^. Trap depth or activation energy (E) is assigned to a metastable state (trap) existing in forbidden band gap region either below the conduction band, above the valence band or rarely in the middle of band gap. These trap states originate from defects in the crystal structure^21^. On irradiating the crystal with a suitable ionizing radiation, free charge carriers are generated which get trapped in these metastable states. On receiving thermal energy, trapped carriers gain much energy to overcome the trap potential barrier and jump to conduction band. Recombination of carriers undergoing transition from conduction band to hole traps lead to TL glow curves. Depending upon the value of *E*, traps may be classified into shallow (for lower values of *E*) and deep traps (for higher *E*). Frequency factor, also known as attempt to escape frequency (s) is number of times per second a charge carrier attempts to escape the potential barrier of trap^21^. So far, there are fewer reports on TL study in undoped and doped ZnAl_2_O_4_ crystal system^26–29^. However, available information is incomplete and TL mechanism is not known completely. Since ZnAl_2_O_4_ crystal system embraces multiple defects being dependent on synthesis conditions, it is expected to exhibit sublime TL response.

Aim of the present study is to gain an insight into effect of ionizing radiation and fuel type on the nature of defects generated and in turn on TL of ZnAl_2_O_4_ nanoparticles. Two types of fuels viz. urea and monoethanolamine (MEA) are used to synthesize ZnAl_2_O_4_ nanoparticles. Indepth TL analysis has been carried out on *γ* and UV irradiated samples to predict the dosimetric application of ZnAl_2_O_4_ nanoparticles. Sample prepared using MEA fuel is scripted as S1 and that using urea is scripted as S2 throughout the manuscript.

## Results and Discussion

Structural, morphological and spectroscopic studies have been done on these samples in a previous work reported elsewhere^6^. X-ray diffraction (XRD) study inferred the formation of single phase ZnAl_2_O_4_ in S1 and a minor secondary phase of *α*-Al_2_O_3_ along with ZnAl_2_O_4_ in S2. Rietveld refinement of XRD patterns confirmed the secondary phase presence in S2 and provided a detailed analysis of ZnAl_2_O_4_ crystal structure. Presence of various defects viz. zinc and aluminium vacancies, Al_*Zn*_ antisite defect, oxygen vacancies and cationic interstitials were indicated by refinement and X-ray absorption near edge spectroscopy. Urbach energy values from absorption spectra quantifying the band tailing and PL emission also pointed towards the existence of various shallow and deep defect states in samples. In order to understand the traps distribution, its dependence on type of ionizing radiation and fuel, further spectroscopic and TL studies have been carried out.

### Raman spectroscopy

**Figure 1:**
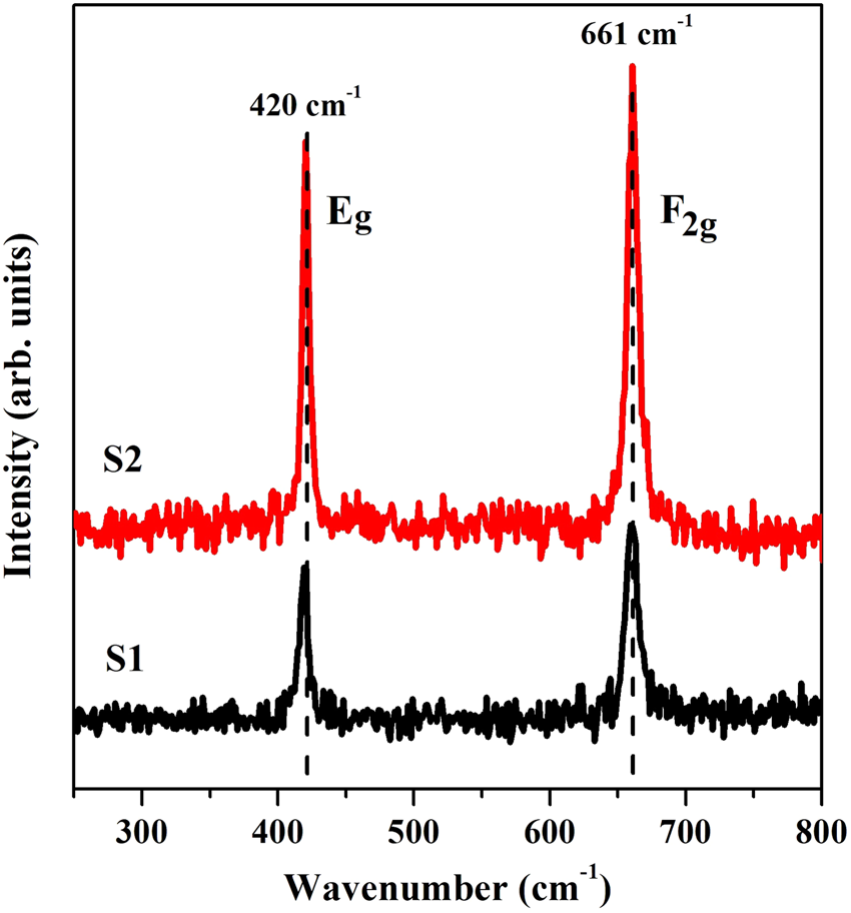
Raman spectra of S1 and S2 showing the signature peak signals of ZnAl_2_O_4_.

Raman spectroscopy has been employed to study the changes in local structure of ZnAl_2_O_4_ nanocrystals on varying the fuel type. Raman spectra for both samples have been shown in Fig. 1. According to group theory, spinels possess 42 normal modes at Brillouin zone center, which may be divided into 3 acoustic and 39 optical modes^30^. These modes can be represented in terms of symmetry species by Eq. (1):

1 $$\Gamma ={A}_{1g}(R)+{E}_{g}(R)+{F}_{1g}(R)+3{F}_{2g}(R)+2{A}_{2u}+2{E}_{u}+5{F}_{1u}(IR)+2{F}_{2u}$$

where, *R* indicates Raman active, *IR* indicates infrared active and remaining are silent modes. *E*_*g*_, *E*_*u*_ and *F*_1*g*_, *F*_2*g*_, *F*_1*u*_, *F*_2*u*_ modes are doubly and triply degenerate, respectively. *F*_1*u*_ species include the acoustic modes. Five modes namely, *A*_1*g*_, *E*_*g*_ and *3F*_2*g*_ are observed in case of spinel structures. However, position and intensity of peaks vary as the cationic species change^30^. Signature peaks of high intensity at 420 cm^−1^ and 661 cm^−1^ correspond to *E*_*g*_ (asymmetric bending motion of oxygen atoms in ZnO_4_ tetrahedra) and *F*_2*g*_ mode (motion of oxygen atoms within AlO_6_ octahedra), respectively. Researchers have observed other modes as well with relatively weak intensities, however in our case only these two prominent modes are observed. This might be an indication of defects present in the system. Low frequency motions (<250 cm^−1^) are mainly due to Zn ions and higher ones are due to O and Al, with dominating contribution from O^31^. Any peak asymmetry indicates the disorder in spinel. It is clear from the figure that peaks corresponding to *F*_2*g*_ mode in both samples are asymmetric, indicating presence of disorder viz. defects in samples^32^. Shoulder peak of *E*_*g*_ mode in both samples is due to bending mode of Al at tetrahedral site thus indicating the existence of AlO_4_ tetrahedra i.e. Al_*Zn*_ antisite defect^30^.

### Scanning and transmission electron microscopy

**Figure 2:**
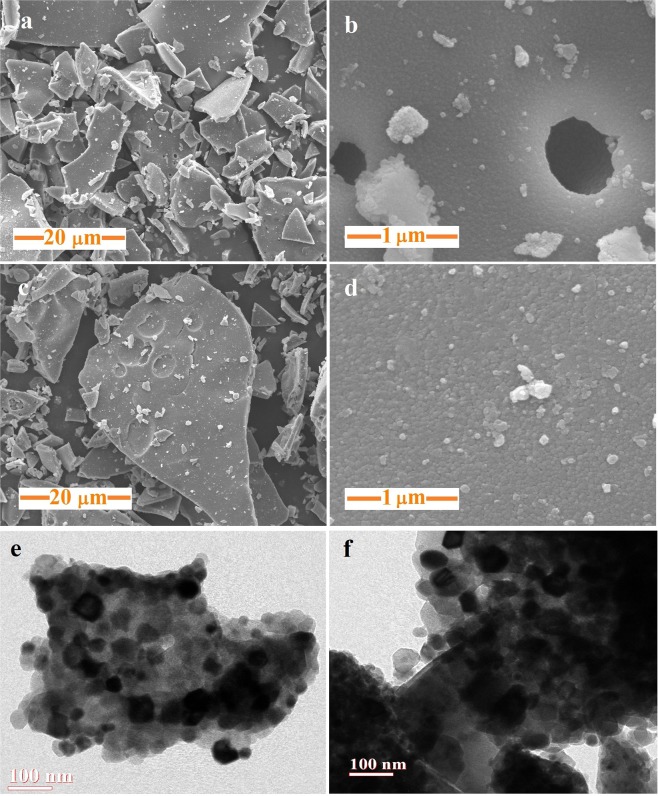
Scanning electron microscopic images of (**a,b**) S1 and (**c,d**) S2 at different resolutions; transmission electron microscopic images of (**e**) S1 and (**f**) S2.

In order to get an idea about surface morphology at a micro scale, scanning electron microscopy (SEM) is used. SEM images of both samples at different resolutions are shown in Fig. 2(a-d). Synthesized nanoparticles have flaky morphology at the surface. Images at higher resolution give a glimpse of agglomerated nanoparticles. Agglomeration may be ascribed to annealing at such a high temperature (1000 °C). Surface morphology of nanoparticles is observed to be approximately spherical with somewhat more particle uniformity in S1 as compared to S2. ImageJ software (version: 1.8.0_112; url: https://imagej.nih.gov/ij/download.html)^33^ is employed to study the particle size from the SEM images and outcome is in the range of 25–35 nm for S1 and 30–50 nm for S2. Transmission electron microscopy (TEM) is also done on both samples to analyze the spatial morphology of nanoparticles. It can be seen from Fig. 2(e,f) that morphology of nanocrystals is nearly spherical with agglomeration. Particle size distribution from TEM images is also analyzed through ImageJ software and it is in the range 20–50 nm and 30–80 nm for S1 and S2, respectively.

### X-ray photoelectron spectroscopy

**Figure 3:**
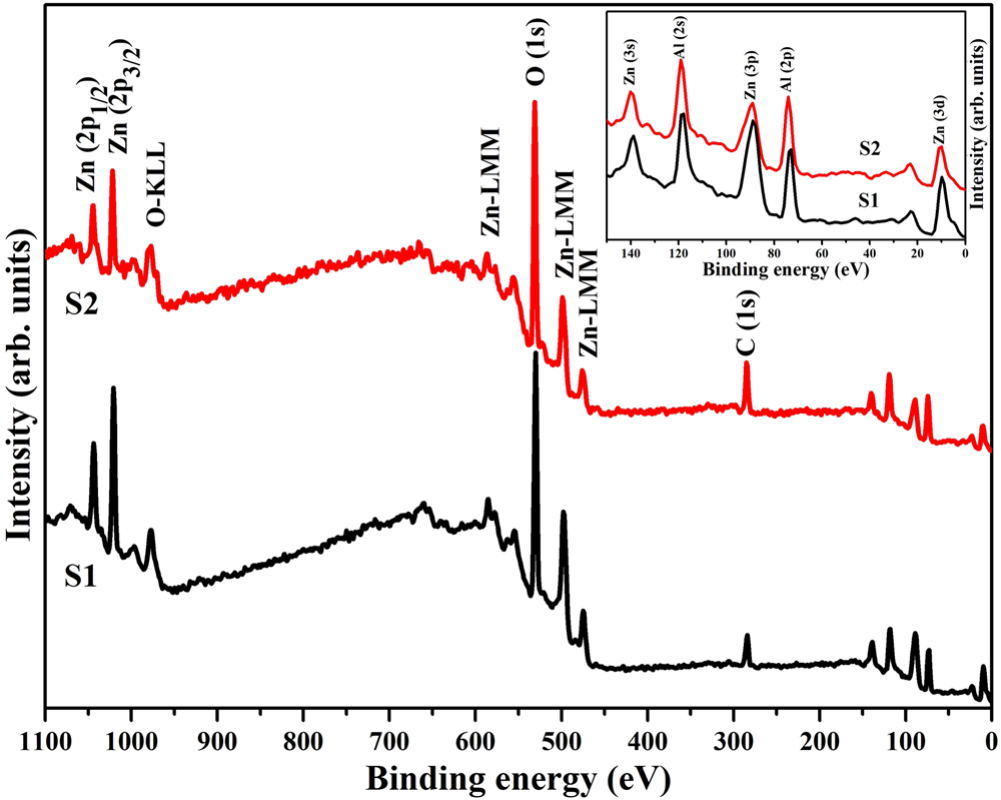
Survey spectra of ZnAl_2_O_4_ nanoparticles. Inset of figure shows the enlarged view of low binding energy region incorporating various Zn and Al orbitals.

The X-ray photoelectron spectroscopy (XPS) survey scans of prepared samples are shown in Fig. 3, which confirm the presence of Zn, Al, O and rule out the incorporation of any other impurity^34^.

**Figure 4:**
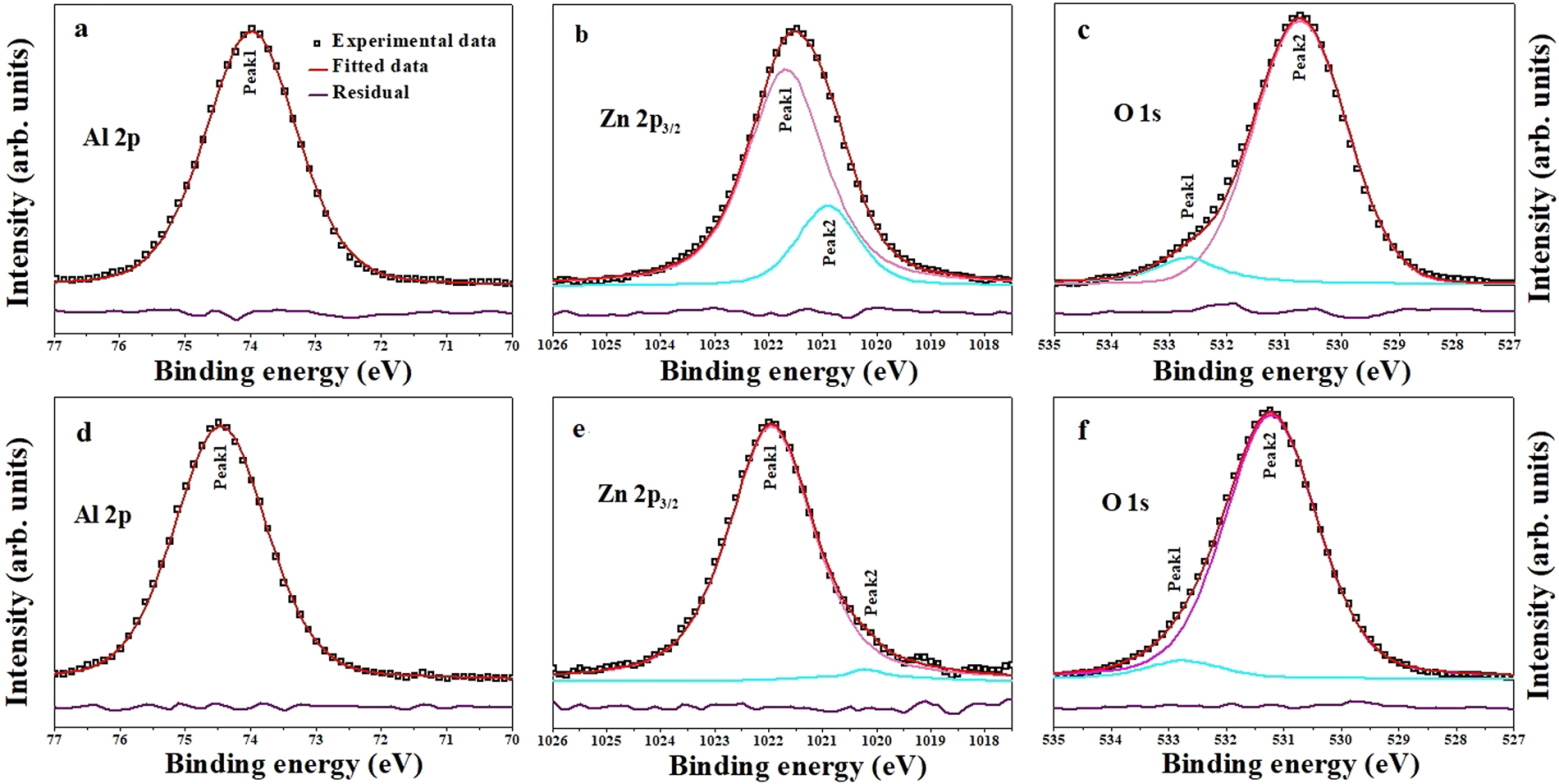
Core level spectra (**a**) Al 2p, (**b**) Zn 2p, (**c**) O 1 s of S1 and (**d–f**) corresponding edges of S2.

Core level spectra of Al 2p, Zn 2p_3/2_ and O 1s are shown in Fig. 4. These spectra are deconvoluted and fitted using Fityk software (version: 1.3.1; url: https://fityk.nieto.pl/)^35^ by applying pseudo-voigt peak profile function. It can be seen that Al 2p peak consist of one peak positioned at 73.98 and 74.46 eV in S1 and S2, respectively. This peak originates due to octahedral position of Al in ZnAl_2_O_4_ lattice i.e. Al in AlO_6_ octahedra^36^. Slightly lower binding energy (B.E.) in S1 can be attributed to presence of few Al^3+^ cations on tetrahedral sites, in other words, Al_*Zn*_ antisite defect. Zn 2p_3/2_ peak is deconvoluted into two components viz. 1021.68 and 1020.90 eV in S1 and 1021.95 and 1020.21 eV in S2. Peak at higher B.E. is due to presence of Zn in tetrahedral sites of ZnAl_2_O_4_ crystal lattice, while peak at lower energy indicates formation of Zn-Zn bond i.e. Zn in metallic form^37^. Relative contribution of peak2 indicates more metallic zinc in S1 as compared to that in S2. Presence of metallic zinc further indicates zinc vacancies in ZnAl_2_O_4_ lattice. O 1s peak is deconvoluted into two peaks with their maxima at 532.68 and 530.72 eV in S1 and 532.75 and 531.22 eV in S2. Peak corresponding to lower B.E. may be ascribed to the oxygen sites in ZnAl_2_O_4_ crystal system, whereas peak at higher B.E. indicates chemisorbed oxygen on sample surface^37^. Shift in low energy peak maximum in S2 towards higher B.E. side is due to the presence of *α*-Al_2_O_3_ as a secondary phase in it, since Al 2p in Al_2_O_3_ exhibits a peak maximum at ~531.6 eV. Information obtained from core level spectra analysis has been summed up in Table 1.

**Table 1:** Information derived from analysis of core level spectra.

Element	Parameter	S1		S2		Reason
		Peak1	Peak2	Peak1	Peak2	
Al 2p	B.E. (eV)	73.98	—	74.46	—	Presence of Al in octahedral sites in ZnAl_2_O_4_ crystal lattice
	Relative contribution	100%		100%		
Zn 2p_3/2_	B.E. (eV)	1021.68	1020.90	1021.95	1020.21	Peak1 due to presence of Zn in tetrahedral voids in ZnAl_2_O_4_ crystal lattice and Peak2 due to Zn metallic clusters
	Relative contribution	78.96%	21.04%	95.88%	4.12%	
O 1s	B.E. (eV)	538.68	530.72	532.75	531.22	Peak1 due to chemisorbed oxygen on sample surface and Peak2 due to oxygen sites in ZnAl_2_O_4_ crystal lattice
	Relative contribution	9.69%	90.31%	12.87%	87.13%	

### Photoluminescence spectroscopy

**Figure 5:**
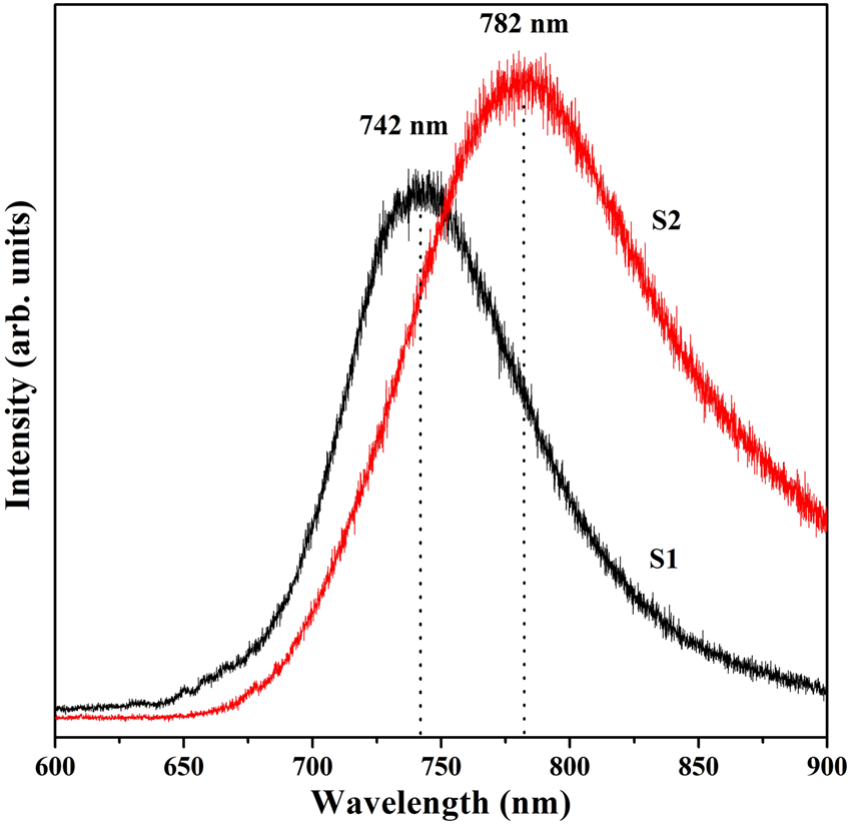
Photoluminescence spectra of S1 and S2 in near-infrared region.

PL spectroscopy can help in probing various defect states present in a compound. PL spectra for both the samples have been shown in Fig. 5. It can be seen that, on excitation with a laser of wavelength 325 nm, a near infrared (NIR) emission results from both samples. However, their intensities and peak positions are not similar. NIR emission is generated due to transitions from defect states and not from band to band ones in ZnAl_2_O_4_. Oxygen vacancies situated deep in forbidden band gap region are responsible for such NIR emission^6^. These defects are excitation energy dependent, as many researchers have earlier observed different emission spectra on changing excitation energy^6,7,9,10,38,39^. Sample S2 is showing a shift of ~40 nm in NIR peak position as compared to S1. This may be attributed to the reordering of radiative defect levels in S2 due to presence of secondary phase. Thus, secondary phase leads emission deeper in NIR region, which could prove beneficial in tissue imaging etc.

### Thermoluminescence

**Figure 6:**
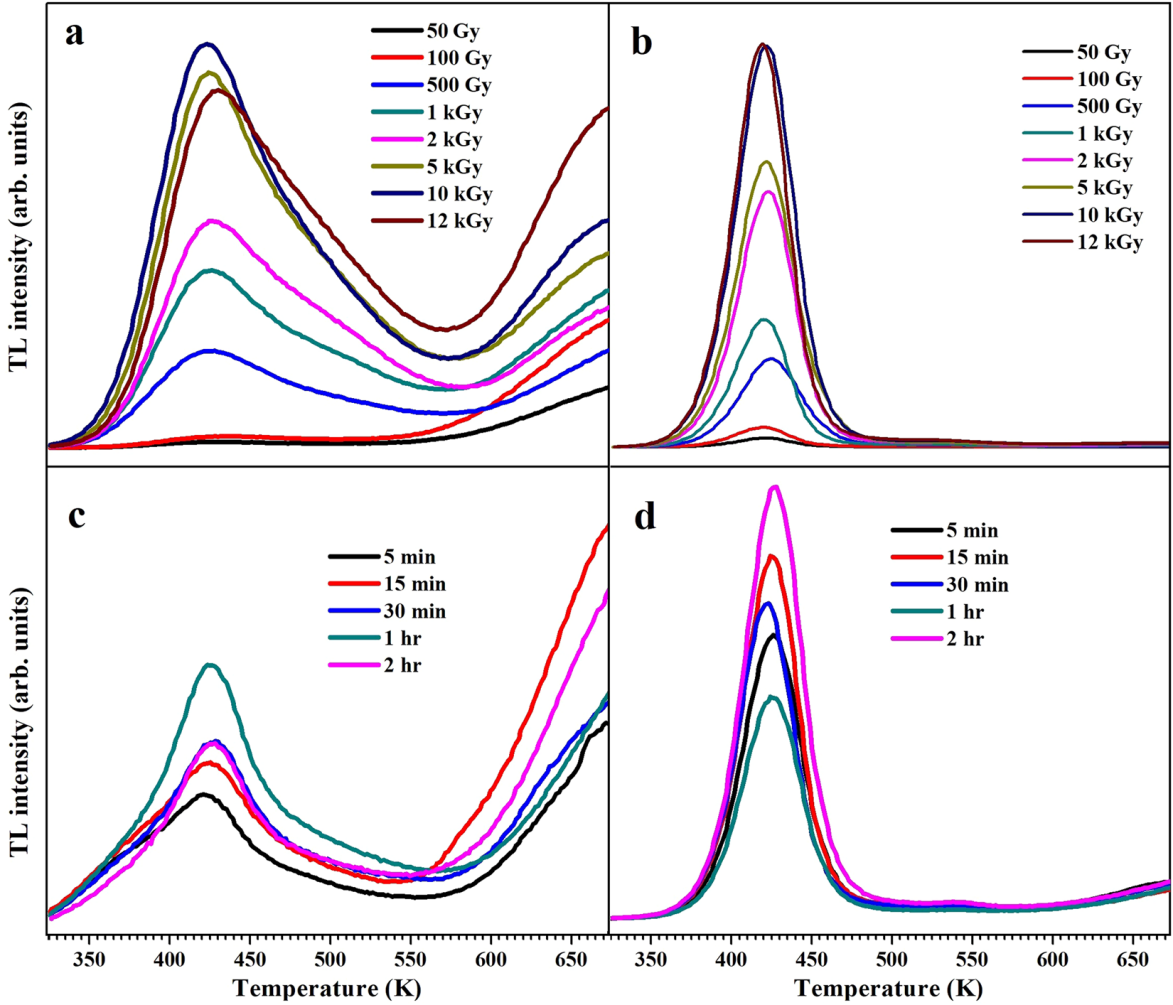
Glow curves of *γ*-irradiated (**a**) S1 (**b**) S2 and UV exposed (**c**) S1 (d) S2.

In order to study various trap states existing within the ZnAl_2_O_4_ crystal system, both the samples were irradiated with varying doses of *γ*-rays and UV radiation. ^60^Co source *γ*-irradiated and UV (254 nm) exposed TL glow curves of both samples are shown in Fig. 6. It can be seen from the Fig. 6(a,c) that S1 shows a broad TL curve with peak maxima at ~426 K on *γ* as well as UV irradiation, while S2 consists of single, narrow and relatively intense peak centered at ~420 K and ~426 K in *γ*-irradiated and UV exposed samples, respectively. TL peak maxima at ~427 K is considered as signature TL signal of ZnAl_2_O_4_ ascribed to O^−^ ions^26,27^. In present case, TL peak maxima is at lower temperature side in *γ*-irradiated S2 (Fig. 6(b)) compared to others which is probably due to the presence of secondary phase in it, since reduced *α*-Al_2_O_3_ exhibits TL peak at 410 K^40^, further indicating oxygen vacancies in the system. Glow curves of S1 are not simple ones, consisting of multiple trap centers which can be acknowledged by deconvolution of the curves. There is almost no change in shape of glow curves of S2 whether irradiated with *γ*-radiation or UV-radiation (Fig. 6(b,d), while a significant change in shape of glow curve occurs in S1 on changing the radiation type (Fig. 6(a,c). This indicates that type of fuel has a significant effect on distribution of defect levels. Glow curves corresponding to S2 have been analyzed by various methods viz. initial rise method (IRM), Chen’s peak shape method (CM) and Kirsh’s method (KM) of whole peak analysis^41^; all applicable for single TL peak.

**Figure 7:**
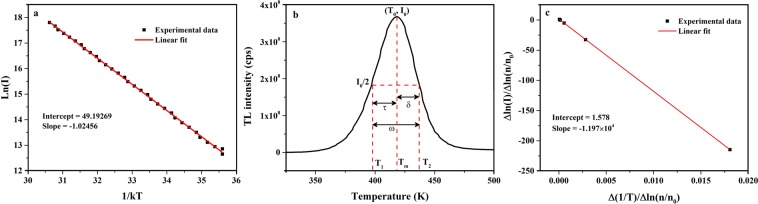
Calculation of kinetic parameters in *γ*-irradiated S2 at 12 kGy from (**a**) Initial rise method, (**b**) Chen’s peak shape method and (**c**) Kirsh’s method.

IRM gives the preliminary estimate of activation energy/trap depth (E) from the analysis of low temperature interval of a peak and is independent of order of kinetics^25^. It is assumed that in low temperature region, amount of trapped carriers is almost constant so temperature dependence can be ignored upto *T*_*c*_ < *T*_*m*_. Here, *T*_*c*_ corresponds to the temperature at which intensity, *I*_*c*_, is less than 15% of maximum intensity, *I*_0_, and *T*_*m*_ is the temperature corresponding to *I*_0_. Under such assumptions, intensity of glow peak can be represented by the Eq. (2):

2 $$I(T)\propto exp(\frac{-\,E}{kT})$$

where, *k* is Boltzmann’s constant. Plot of *ln(I)* as a function of *1/T* gives a straight line, whose slope determines the trap depth as depicted in Fig. 7(a). *E* values for *γ*-irradiated and UV exposed S2 are listed in Tables 2 and 3, respectively.

**Table 2:** TL parameters of *γ*-irradiated S2, derived from initial rise method, Chen’s peak shape method and Kirsh’s method.

Method	Dose	100 Gy	500 Gy	1 kGy	2 kGy	5 kGy	10 kGy	12 kGy
Initial rise method	E (eV)	0.987	1.022	1.038	1.030	1.023	1.030	1.025
Chen’s peak shape method	*τ*	21.15	23.23	21.81	22.04	22.72	22.66	21.30
	*δ*	19.52	20.55	18.02	19.08	20.65	20.14	18.66
	*ω*	40.67	43.78	39.83	41.12	43.37	42.80	39.96
	*μ*_*g*_	0.480	0.469	0.452	0.464	0.476	0.470	0.467
	b	1.52	1.35	1.24	1.38	1.48	1.44	1.36
	E_*τ*_ (eV)	1.075	0.977	0.993	1.014	0.996	0.988	1.042
	E_*δ*_ (eV)	1.094	1.010	1.020	1.042	1.025	1.018	1.067
	E_*ω*_ (eV)	1.091	0.999	1.013	1.034	1.016	1.009	1.061
	s (s^−1^)	5.16 × 10^12^	2.42 × 10^11^	5.06 × 10^11^	7.94 × 10^11^	5.13 × 10^11^	4.20 × 10^11^	2.17 × 10^12^
Kirsh’s method	E (eV)	1.031	1.035	1.043	1.038	1.060	1.023	1.047
	b	1.58	1.74	1.61	1.66	1.92	1.50	1.63
	s (s^−1^)	9.23 × 10^11^	6.83 × 10^11^	1.23 × 10^12^	9.11 × 10^11^	1.83 × 10^12^	6.30 × 10^11^	1.48 × 10^12^

**Table 3:** TL parameters of 254 nm UV-irradiated S2, derived from initial rise method, Chen’s peak shape method and Kirsh’s method.

Method	Irradiation time	5 min	15 min	30 min	1 hr	2 hr
Initial rise method	E (eV)	0.874	0.864	0.877	0.834	0.850
Chen’s peak shape method	*τ*	22.40	21.58	21.17	21.47	22.92
	*δ*	20.87	19.31	19.32	21.89	19.48
	*ω*	43.27	40.89	40.49	43.36	42.40
	*μ*_*g*_	0.482	0.472	0.477	0.505	0.459
	b	1.54	1.44	1.52	1.78	1.31
	E_*τ*_ (eV)	1.048	1.070	1.088	1.134	0.991
	E_*δ*_ (eV)	1.071	1.094	1.108	1.131	1.022
	E_*ω*_ (eV)	1.066	1.089	1.105	1.139	1.012
	s (s^−1^)	1.49 × 10^12^	2.96 × 10^12^	5.78 × 10^12^	1.30 × 10^13^	2.82 × 10^11^
Kirsh’s method	E (eV)	0.951	0.968	0.989	0.939	0.978
	b	1.92	1.84	1.66	1.35	1.77
	s (s^−1^)	6.05 × 10^10^	9.95 × 10^10^	2.18 × 10^11^	4.52 × 10^11^	1.13 × 10^11^

CM can be applied to a well resolved single peak, without any prior knowledge of frequency factor (s) and kinetics order^42^. As the name indicates, this method is based upon analysis of peak shape parameters determining the trap depth and order of kinetics. Herein, T_1_ and T_2_ are the temperature values corresponding to I_0_/2 on lower and higher temperature side, respectively. Peak shape parameters (illustrated in Fig. 7(b)) are: *τ* = *T*_*m*_ − *T*_1_ is half width of peak at low temperature side, *δ* = *T*_2_ − *T*_*m*_ is half width of peak at high temperature side, *ω* = *T*_2_ − *T*_1_ is full width at half maximum, *μ*_*g*_ = *δ*/*ω* is the geometrical form factor. Value of *μ*_*g*_ determines the order of kinetics, for it being 0.42 indicates first order kinetics, 0.52 indicates second order kinetics and value lying in the range 0.42–0.52 indicates mixed order kinetics. The mathematical expression, for general order kinetics, used to calculate trap depth energies is given by Eq. (3) as:

3 $${E}_{\alpha }={c}_{\alpha }(\frac{k{T}_{m}^{2}}{\alpha })-{b}_{\alpha }(2k{T}_{m})$$

where *α* = *τ*, *δ*, *ω* and

$$\begin{array}{rcl}{c}_{\tau } & = & 1.510+3.0({\mu }_{g}-0.42),{b}_{\tau }=1.58+4.2({\mu }_{g}-0.42)\\ {c}_{\delta } & = & 0.976+7.3({\mu }_{g}-0.42),{b}_{\delta }=0\\ {c}_{\omega } & = & 2.52+10.2({\mu }_{g}-0.42),{b}_{\omega }=1\end{array}$$

Value of kinetic order, b can be calculated through *μ*_*g*_ from the graph proposed by Chen^42^. All peak shape parameters, corresponding trap depth energies and order of kinetics computed for S2 are summed up in Tables 2 and 3.

KM is a curve fitting method, an extension of IRM to the whole curve converting it into a straight line^24^. This method can simultaneously provide activation energy and order of kinetics for an isolated peak, based on following mathematical expression:

4 $$\frac{\Delta lnI}{\Delta ln(n/{n}_{0})}=b-\frac{E}{k}.\frac{\Delta ln(1/T)}{\Delta ln(n/{n}_{0})}$$

where, *n*_0_ and *n* are the charge carrier concentration initially and at any temperature (T), respectively. Ratio *n*/*n*_0_ is equal to the area of the peak from point (T, I) till end point. Slope of above straight line gives (−*E*/*k*) and intercept gives *b*, shown in Fig. 7(c). Information obtained from KM for S2 is tabulated in Tables 2 and 3. Attempt to escape frequency or frequency factor (s) can be obtained by applying Eq. (5) for general order kinetics:

5 $$s=\frac{\beta E}{k{T}_{m}^{2}(1+\frac{2k{T}_{m}(b-1)}{E})}exp(\frac{E}{k{T}_{m}})$$

here, *β* is the linear heating rate. Calculated frequency factors in S2 at different doses of *γ* radiation and different exposure times of UV radiation are summed up in Tables 2 and 3, respectively.

**Figure 8:**
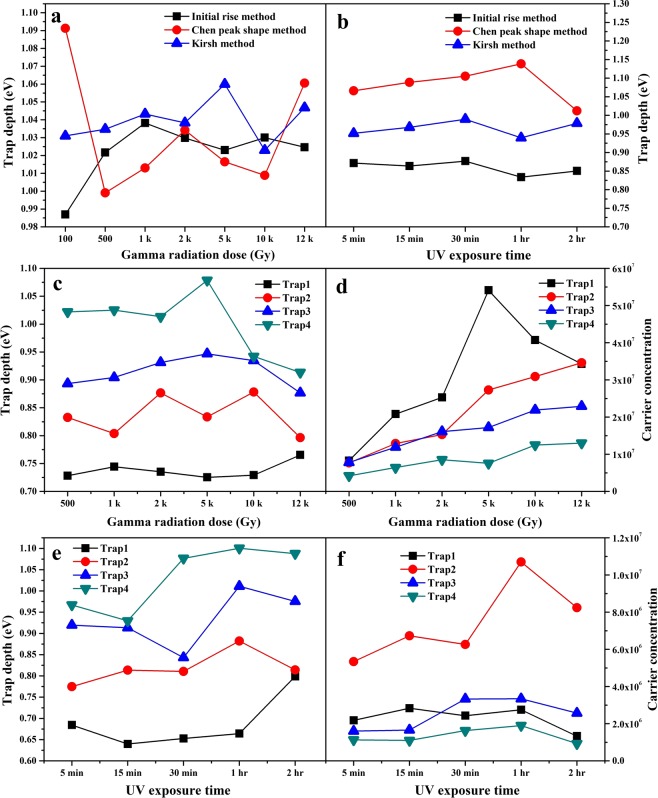
Comparison of trap depths in S2 calculated by different methods at (**a**) various doses of *γ*-irradiation and (**b**) different exposure durations of 254 nm UV radiation; (**c**) trap depths and (**d**) carrier concentration of various traps in S1 at various *γ*-radiation doses; (**e**) trap depths and (**f**) carrier concentration of various traps in S1 at various UV exposure durations.

All three methods indicate trap depth in S2 to be in the range 0.9–1.1 eV and glow curves following a general order kinetics. IRM underestimates the trap depths due to inclusion of non radiative emission as well in computation^25^. Kirsh’s method being a whole curve analysis method can be relied on the most. Relative comparison in results of all three methods is shown graphically in Fig. 8.

**Figure 9:**
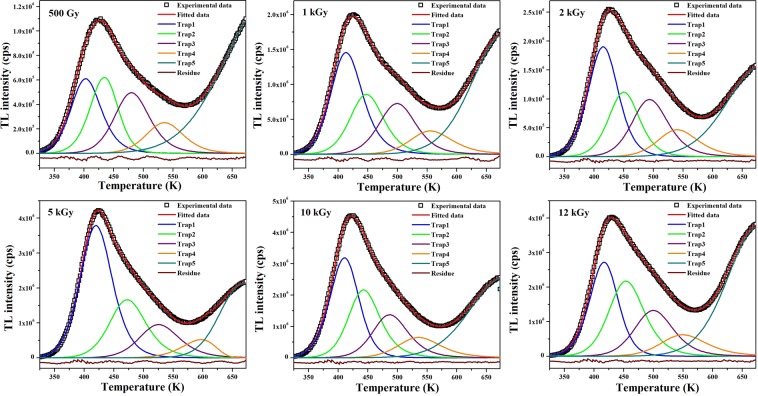
Computerized deconvoluted glow curves of S1 at different doses of *γ*-radiation.

**Figure 10:**
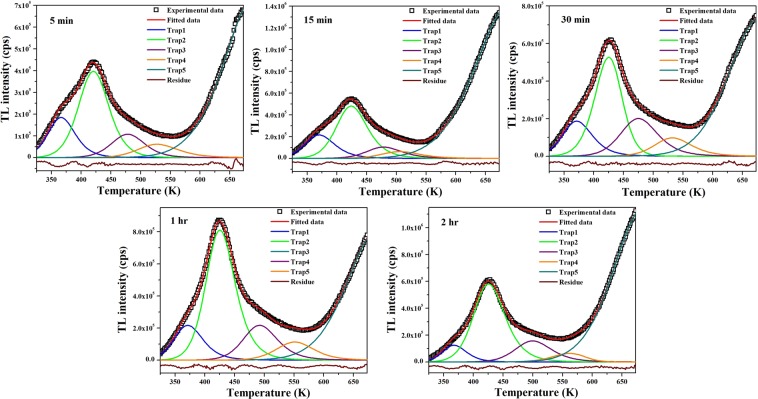
Computerized deconvoluted glow curves of S1 at different exposure durations of UV-radiation.

Methods applied for the analysis of glow curves of S2 can’t be applied for the same in S1, due to overlapped peaks. So, information from glow curves of S1 has been derived by deconvoluting the curves on applying general order kinetics in TLanal software (version: 1.0.3; url: http://physica.gsnu.ac.kr/TLanal/start.html)^43^ specially meant for deconvoluting complex TL curves^44^. Deconvoluted glow curves are shown in Figs. 9 and 10, respectively and glow curve parameters are summed up in Tables 4 and 5. Deconvolution enabled to visualize the trap distribution and trap depths. Population of trapped carriers is in decreasing order as one moves from lower temperature side to higher one for all *γ*-doses and UV exposure durations. Frequency factors are relatively lower than those in S2. Retrapping phenomenon plays its role where the trapping states are closely situated. The trap states may be so closely lying that their charge wavefunctions may overlap and tunneling occurs among trap centers. This cause retrapping of charge carriers released from shallower traps to deeper ones and hence lowers the frequency factors^20^. Goodness of fit has been judged by calculating the figure of merit (FOM) as per relation 6:

6 $$FOM=\frac{\Sigma |T{L}_{Experimental}-T{L}_{Fitted}|}{\Sigma |T{L}_{Fitted}|}$$

**Table 4:** TL parameters for different traps derived from computerized deconvolution of *γ*-irradiated S1.

Dose	Peak	T_m_ (K)	I_0_	n_0_	E(eV)	s (s^−1^)	b	FOM
500 Gy	Peak1	402.49	6.09 × 10^5^	8.28 × 10^6^	0.7282	3.15 × 10^8^	1.8768	1.150
	Peak2	434.24	6.21 × 10^5^	7.67 × 10^6^	0.8328	1.13 × 10^9^	1.5385	
	Peak3	480.01	4.95 × 10^5^	7.80 × 10^6^	0.8933	5.02 × 10^8^	1.8615	
	Peak4	535.80	2.51 × 10^5^	4.21 × 10^6^	1.0220	8.00 × 10^8^	1.7846	
1 kGy	Peak1	413.26	1.46 × 10^6^	2.08 × 10^7^	0.7443	2.81 × 10^8^	1.9385	1.185
	Peak2	447.47	8.59 × 10^5^	1.29 × 10^7^	0.8039	2.46 × 10^8^	1.8367	
	Peak3	500.35	7.27 × 10^5^	1.19 × 10^7^	0.9044	2.64 × 10^8^	1.7979	
	Peak4	555.43	3.39 × 10^5^	6.42 × 10^6^	1.0252	3.58 × 10^8^	2.0000	
2 kGy	Peak1	415.23	1.90 × 10^6^	2.53 × 10^7^	0.7353	1.96 × 10^8^	1.6605	0.822
	Peak2	450.58	1.12 × 10^6^	1.53 × 10^7^	0.8768	1.55 × 10^9^	1.7650	
	Peak3	493.24	9.89 × 10^5^	1.61 × 10^7^	0.9313	6.72 × 10^8^	1.9140	
	Peak4	539.96	4.66 × 10^5^	8.54 × 10^6^	1.0134	5.34 × 10^8^	2.0000	
5 kGy	Peak1	419.91	3.79 × 10^6^	5.41 × 10^7^	0.7253	1.41 × 10^8^	1.7716	1.136
	Peak2	472.26	1.66 × 10^6^	2.73 × 10^7^	0.8337	1.57 × 10^8^	1.8923	
	Peak3	525.50	9.54 × 10^5^	1.72 × 10^7^	0.9469	2.23 × 10^8^	1.9077	
	Peak4	597.12	5.20 × 10^5^	7.56 × 10^6^	1.0786	2.22 × 10^8^	1.0308	
10 kGy	Peak1	410.64	3.19 × 10^6^	4.07 × 10^7^	0.7292	2.12 × 10^8^	1.5894	0.729
	Peak2	442.80	2.17 × 10^6^	3.09 × 10^7^	0.8782	2.39 × 10^9^	2.0000	
	Peak3	486.47	1.38 × 10^6^	2.19 × 10^7^	0.9347	1.01 × 10^9^	1.9538	
	Peak4	535.40	6.56 × 10^5^	1.25 × 10^7^	0.9422	1.30 × 10^8^	2.0000	
12 kGy	Peak1	416.77	2.72 × 10^6^	3.43 × 10^7^	0.7654	1.37 × 10^8^	1.5942	0.658
	Peak2	453.74	2.17 × 10^6^	3.46 × 10^7^	0.7965	1.47 × 10^8^	1.9077	
	Peak3	499.91	1.32 × 10^6^	2.29 × 10^7^	0.8768	1.31 × 10^8^	1.8615	
	Peak4	549.50	6.12 × 10^5^	1.30 × 10^7^	0.9134	3.83 × 10^7^	2.0898

**Table 5:** TL parameters for different traps derived from computerized deconvolution of UV-irradiated S1.

Exposure time	Peak	T_m_ (K)	I_0_	n_0_	E(eV)	s (s^−1^)	b	FOM
5 min	Peak1	366.28	1.85 × 10^5^	2.17 × 10^6^	0.6843	6.27 × 10^8^	1.9077	1.881
	Peak2	420.43	3.97 × 10^5^	5.34 × 10^6^	0.7747	4.57 × 10^8^	1.7639	
	Peak3	480.01	1.07 × 10^5^	1.59 × 10^6^	0.9195	1.06 × 10^9^	1.7854	
	Peak4	535.80	6.15 × 10^4^	1.13 × 10^6^	0.9670	3.23 × 10^8^	2.0000	
15 min	Peak1	369.84	2.15 × 10^5^	2.83 × 10^6^	0.6397	1.24 × 10^8^	2.0000	1.350
	Peak2	423.96	4.80 × 10^5^	6.73 × 10^6^	0.8135	1.13 × 10^9^	1.9732	
	Peak3	478.84	1.04 × 10^5^	1.66 × 10^6^	0.9133	8.99 × 10^8^	2.0000	
	Peak4	506.03	6.31 × 10^4^	1.10 × 10^6^	0.9296	3.54 × 10^8^	2.0000	
30 min	Peak1	370.98	1.87 × 10^5^	2.43 × 10^6^	0.6526	1.76 × 10^8^	2.0000	1.385
	Peak2	425.23	5.27 × 10^5^	6.26 × 10^6^	0.8106	1.01 × 10^9^	1.4769	
	Peak3	474.88	2.00 × 10^5^	3.33 × 10^6^	0.8431	1.76 × 10^8^	1.9231	
	Peak4	532.46	9.73 × 10^4^	1.63 × 10^6^	1.0768	3.23 × 10^9^	1.9692	
1 hr	Peak1	371.04	2.17 × 10^5^	2.75 × 10^6^	0.6642	2.58 × 10^8^	1.9692	1.695
	Peak2	425.49	8.09 × 10^5^	1.07 × 10^7^	0.8820	7.43 × 10^9^	2.0000	
	Peak3	492.28	2.17 × 10^5^	3.34 × 10^6^	1.0110	5.09 × 10^9^	2.0000	
	Peak4	550.98	1.13 × 10^5^	1.90 × 10^6^	1.1005	2.28 × 10^9^	1.8461	
2 hr	Peak1	366.96	1.25 × 10^5^	1.33 × 10^6^	0.7988	2.82 × 10^10^	2.0000	1.672
	Peak2	425.38	5.80 × 10^5^	8.24 × 10^6^	0.8142	1.06 × 10^9^	2.0000	
	Peak3	499.48	1.58 × 10^5^	2.57 × 10^6^	0.9754	1.48 × 10^9^	2.0000	
	Peak4	561.68	6.43 × 10^4^	9.87 × 10^5^	1.0879	1.10 × 10^9^	1.2923	

where, *TL*_*Experimental*_ and *TL*_*Fitted*_ are the intensity values of experimental and fitted data, respectively. FOM must be minimized to achieve best fit and reliable parameters. Its value should be within a tolerance range of 0–3%^41^. Relative comparison of trap depth and carrier concentration of various traps at different doses of *γ* radiation and different UV exposure durations in S1 is shown in Fig. 8.

Incomplete glow curve at higher temperature side exhibited by S1 (Fig. 6(a,c)) is generally observed in ZnAl_2_O_4_ due to F^+^ defect centers^26^. This means sample S1 contains F^+^ centers which are dominant at lower doses of *γ* radiation and for all exposure times of UV irradiation. As the gamma dose increases, shallow traps (F centers) become more populated as compared to deep traps (F^+^ centers). However, saturation reaches at 10 kGy, beyond which population of carriers in F^+^ centers are competing with that in shallow traps. Since intensity of a glow curve is related to the population of trapped carriers, so by comparing intensities at different doses and exposure times, one can obtain information about the changes in carrier concentration of trap centers. With the increase in *γ*-dose, intensity increased which mean trap carrier concentration increased. This is due to increased filling of traps with electrons and increased number of photons emitted on recombination. In S1, saturation occurs at 10 kGy dose and no more traps could be filled by further increasing the dose, however, a minimal increase in carrier concentration is achieved in S2 with increase in dose beyond 10 kGy. This could be explained with the help of track interaction model^19^. According to this model, population of free charge carriers generated on irradiation depend upon the cross-section of tracks and length of tracks in lattice. Length of tracks is limited to few tens of nanometers in nanostructures, so fewer trap centers are present for lower doses. Saturation occurs on increasing dose when track overlapping occurs resulting in generation of no new free carriers. However, if particle size is further reduced, leading to higher surface-to-volume ratio, some particles missed at lower doses would be encountered by higher doses leading to increased TL intensity. S1 is smaller in size as compared to S2, difference being ~10 nm, so saturation does not occur at 10 kGy in S1 in accordance with track interaction model.

Samples were also exposed to UV radiation at 365 nm (see Supplementary Fig. S1) and ^137^Cs source *γ*-radiation for 14 hour (see Supplementary Fig. S2). No TL signal was observed in lower temperature range, but incomplete glow curve at higher temperature side was visible in both S1 and S2 for both type of irradiation exposures. This means samples and hence trap levels are sensitive to type of ionizing radiation. F^+^ centers originate when deeper traps transfer charge to them. Higher temperature glow curve also known as spurious TL is not supposed to be generating from irradiation. However, irradiation fosters the charge transfer from deeper defects to F^+^ centers. Thus, type of incident radiation has a significant effect on activating the trap states and their population.

**Figure 11:**
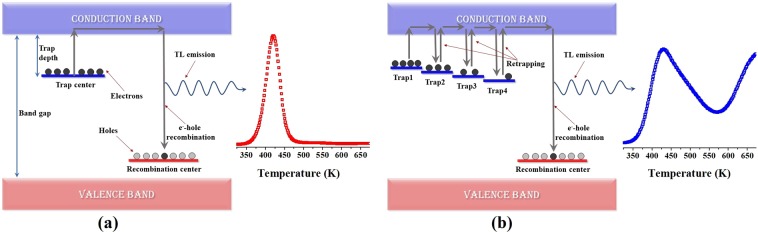
Schematic of thermoluminescence emission in (**a**) S2 and (**b**) S1.

Traps at lower temperature side corresponds to shallow traps i.e. charge carriers require lesser energy to get out of them and those at higher temperature sides correspond to deeper traps. Since in S1, intensity of low temperature peak is lesser and broader as compared to S2, it might be concluded that carriers from shallow traps are transferred to the deeper traps. Also, traps in S1 are continuously distributed, whereas only single trap state is present in S2, causing the broadening of peak in S1. Schematic of mechanism of trapping, retrapping and recombination in S1 and S2 is shown in Fig. 11. As reported in previous work, forbidden band gap of both samples contain multiple defect levels, which are further distributed into near band edge defects and defects deeper in forbidden region^6,7^. Defect levels closer to valence band edge may correspond to hole traps or V centers. Thus, Zn or Al vacancies being located near valence band edge might serve as hole traps. Whereas, oxygen vacancies form F and F^+^ centers by trapping electrons and are present near conduction band edge. Presence of these defects near valence band edge, conduction band edge and deeper in band gap have been also confirmed by various researchers through ab-initio calculations thereby supporting the presented results^9,12,45^.

In order to check the effect of *γ* and UV irradiation on the luminescence of both samples, PL has been conducted on them after irradiation. PL spectra of S1 and S2 irradiated at various *γ* radiation doses are reported in Supplementary Information (Figs. S3 and S4, respectively) and corresponding spectra of S1 and S2 exposed to UV radiation for different duration are reported in supplementary information (Fig. S5 and S6, respectively). It is observed from the spectra that there is no significant effect of *γ*-irradiation and UV exposure on the PL emission of both the samples. Thus, it can be concluded that the defects activated by *γ* or UV irradiation participate in TL whilst not in PL in samples.

## Conclusions

Different fuels, particularly urea and MEA, are employed for combustion synthesis of ZnAl_2_O_4_ nanoparticles. Prepared nanoparticles have already been characterized through various spectroscopic techniques indicating simultaneous presence of multiple defects. Thermoluminescence study have been utilized to further enhance the understanding of defect levels. TL response curves of samples irradiated at varying *γ*-radiation doses and different UV exposure times have inferred the presence of O^−^ defects and F^+^ centers. It has been observed that one trap-one recombination model is applicable to S2, while multiple traps are present in S1. Retrapping phenomenon is expected to be playing its role in S1, leading to lowering of frequency factors and a wide distribution of charge carriers in all traps as compared to high frequency factor and charge carriers densely populated in one type of trap in S2. Trap distribution, trapping-retrapping and recombination phenomenon is depicted through a schematic band model. XPS has helped to analyze the bonding environment of Zn, Al and O elements in prepared samples. Presence of dual phase in S2, Zn metallic clusters, Zn vacancies, Al_*Zn*_ defects, oxygen vacancies in both samples is also confirmed through core level spectra analysis supporting the results by TL. PL and Raman spectroscopy have also indicated the presence of Al_*Zn*_ antisite defect and oxygen vacancies in both systems. SEM and TEM images depict the agglomerated nature of the spherical nanoparticles forming sheets/flakes type morphology on the surface. Presented study aids the fundamental information of defect states in ZnAl_2_O_4_ lattice and their probable contribution in various properties. It can be summed up that ZnAl_2_O_4_ with a minor phase of Al_2_O_3_ can serve as an excellent candidate for dosimetry.

## Methods

### Synthesis

ZnAl_2_O_4_ nanoparticles were synthesized through combustion method. Zinc nitrate hexahydrate (Zn(NO_3_)_2_.6H_2_O) and aluminium nitrate nonahydrate (Al(NO_3_)_3_.9H_2_O) were used as oxidizers. Two different fuels namely urea (CO(NH_2_)_2_) and MEA (C_2_H_7_NO) were utilized for combustion. Experimental procedure for synthesis of samples is explained in detail elsewhere^6^.

### Characterization

In order to examine the properties of prepared nanoparticles, various characterization techniques have been used. Raman spectroscopy is carried out on both samples using Ar laser 540 nm in Renishaw IN-VIA Raman microscope. Scanning electron microscopy has been used to study surface morphology of the samples at different resolutions through SEM, FEI XL-30 FEG. Transmission electron microscopy of the samples is performed on Tecnai 200 keV by FEI. Elemental confirmation and chemical state information have been derived through X-ray photoelectron spectroscopy from PHI-5000 VersaProbe scanning XPS microprobe (Ulvac-PHI, Japan) using monochromatised Al K_*α*_ (1486.6 eV). The charging of samples during the X-ray irradiation was taken care of by neutralizing with slow electrons and ions during the data acquisition. The charge over compensation resulting in shifts in the measured binding energies was corrected using adventitious C 1s peak (284.6 eV). The sample area analyzed was about 0.1 mm^2^ and the pressure during acquisition was less than 2 × 10^−7^ Pa. Photoluminescence spectroscopy has been employed to study the defect-induced luminescence using Renishaw IN-VIA Raman microscope at an excitation wavelength of 325 nm using HeCd laser. Harshaw TL reader, model 3500 has been used to study TL response of samples pre-annealed at 400 °C and at different doses of *γ*-radiation and UV radiation for different durations. ^60^Co source has been used in gamma chamber 1200 for irradiation at different doses specifically 50 Gy, 100 Gy, 500 Gy, 1 kGy, 2 kGy, 5 kGy, 10 kGy and 12 kGy. TL is also recorded for samples irradiated for 14 hour with *γ*-rays from ^137^Cs source placed in a lead block. UV lamp, model VL-6.LC, used to expose samples for different durations (5 min, 15 min, 30 min, 1 hour, 2 hour) is equipped with two wavelengths (254 nm (6W) and 365 nm (6W)). TL signal has been monitored in 50–400 °C temperature range at a constant heating rate of 5 °C/s.

## Supplementary information

Supplementary information.
