# Supplementary material for: Defect states and kinetic parameter analysis of ZnAl2O4 nanocrystals by X-ray photoelectron spectroscopy and thermoluminescence
Source: Sci Rep. 2020 Jan 15;10:385. doi: 10.1038/s41598-019-57227-8 (PMC6962157; doi:10.1038/s41598-019-57227-8)
Supplement: Supplementary file 1 — Supplementary information. [file 41598_2019_57227_MOESM1_ESM.pdf]

Defect states and kinetic parameter analysis of $ZnAl_2O_4$ nanocrystals by X-ray photoelectron spectroscopy and thermoluminescence

Megha Jain[1,2], Manju[1, 2], Ravi Kumar[3], Sung Ok Won[4], Keun Hwa Chae[4], Ankush Vij[5,†], and Anup Thakur[1,*]

[1]Punjabi University, Advanced Materials Research Lab, Department of Basic and Applied Sciences, Patiala, 147 002, India
[2]Punjabi University, Department of Physics, Patiala, 147 002, India
[3]National Institute of Technology, Center for Material Science & Engineering, Hamirpur, 177 005, India
[4]Korea Institute of Science and Technology, Advanced Analysis Center, Seoul, 02792, South Korea
[5]Amity University Haryana, Nanophosphors Lab, Department of Applied Physics, Gurgaon, 122 413, India
[†]corresponding.vij_anx@yahoo.com
[*]corresponding.dranupthakur@gmail.com

ABSTRACT

Defect states in $ZnAl_2O_4$ have a significant role in its applicability as a luminescent material. To understand the nature and distribution of defects in its crystal lattice, thermoluminescence (TL) study has been carried out. Excellent TL response is observed for γ- and ultraviolet-irradiated samples at different doses and durations, respectively. Different types of fuel employed in combustion synthesis show a remarkable effect on the trap distribution and hence luminescence properties. Shallow and deep traps are observed in crystals attributed to O^- vacancies and F^+ centers. The mechanism of trapping, retrapping and recombination have been depicted through schematic band model diagram. X-ray photoelectron spectroscopy indicated the presence of various types of defects specifically Al_{Zn} antisite defect, oxygen and zinc vacancies which are further upheld by photoluminescence and Raman spectroscopy. All results when summed up, predict $ZnAl_2O_4$ to be a quality material for dosimetry.

Supplementary information

Supplementary Fig. S1: Glow curves of S1 and S2 samples, irradiated with UV rays of 365 nm wavelength for different durations.
Supplementary Fig. S2: Glow curves of S1 and S2 samples, irradiated with ^{137}Cs γ ray for a duration of 14 hours.
Supplementary Fig. S3: PL spectra of sample S1 irradiated with different γ radiation doses.
Supplementary Fig. S4: PL spectra of sample S2 irradiated with different γ radiation doses.
Supplementary Fig. S5: PL spectra of sample S1 exposed with UV radiation for different time periods.
Supplementary Fig. S6: PL spectra of sample S2 exposed with UV radiation for different time periods.

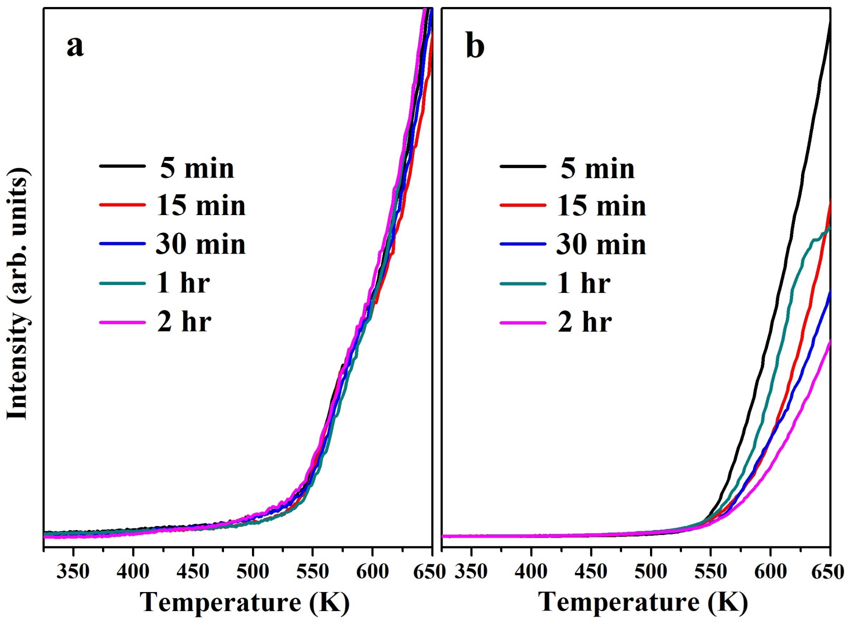

Figure S1. TL glow curves of (a) S1 and (b) S2 at 365 nm UV-irradiation for different durations.

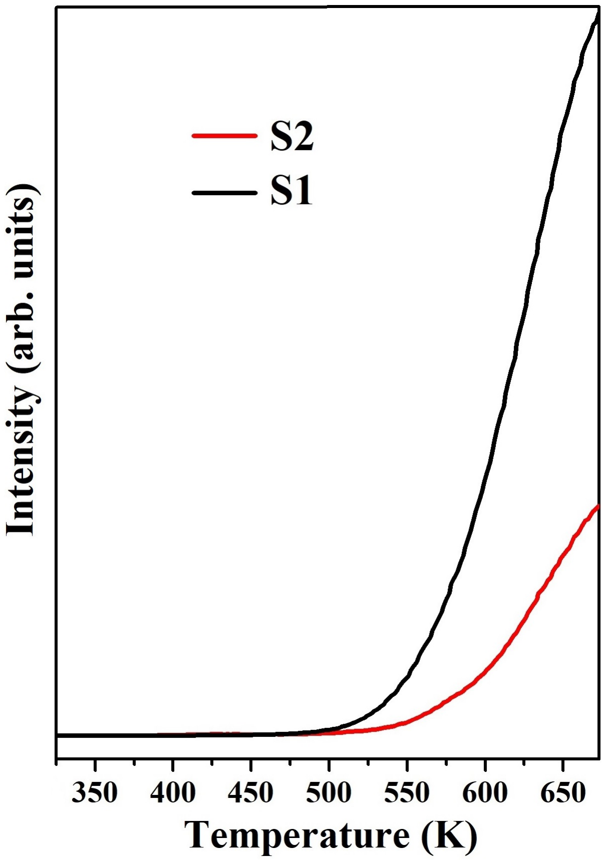

Figure S2. TL glow curves of S1 and S2 irradiated with γ ray from ^{137}Cs source for 14 hour.

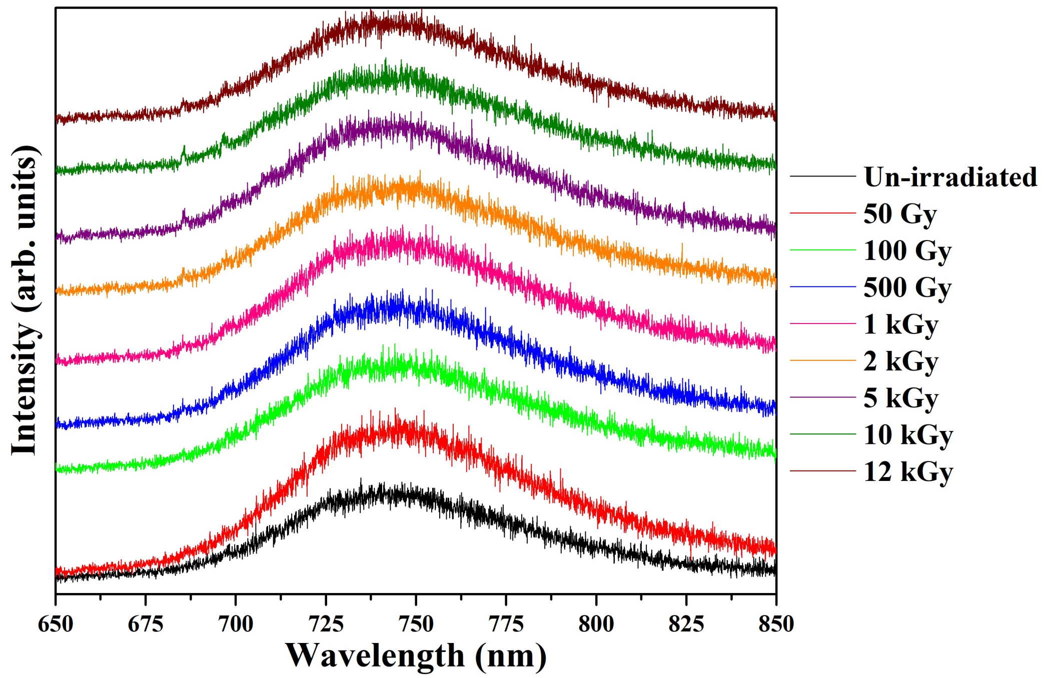

Figure S3. PL spectra of S1 irradiated at different γ doses.

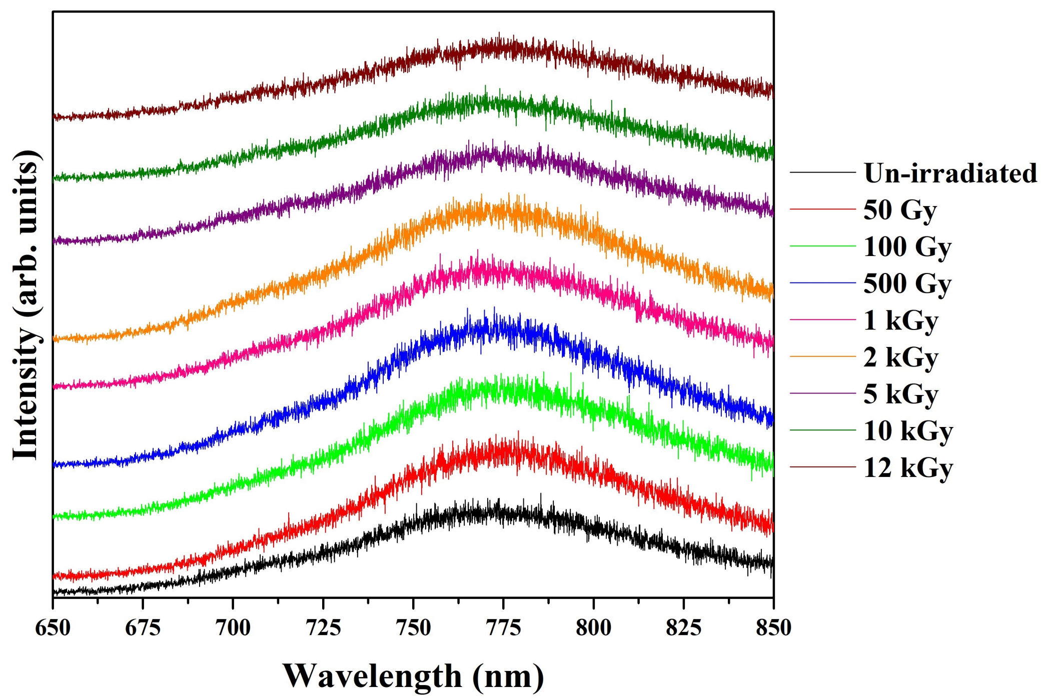

Figure S4. PL spectra of S2 irradiated at different γ doses.

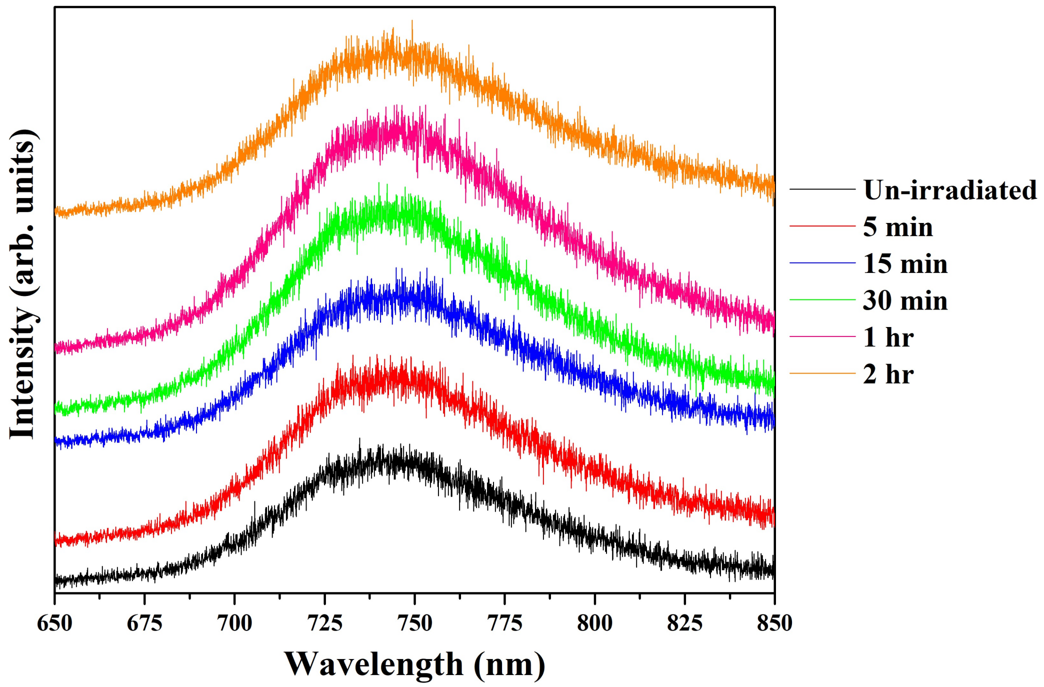

Figure S5. PL spectra of S1 exposed with UV for different duration.

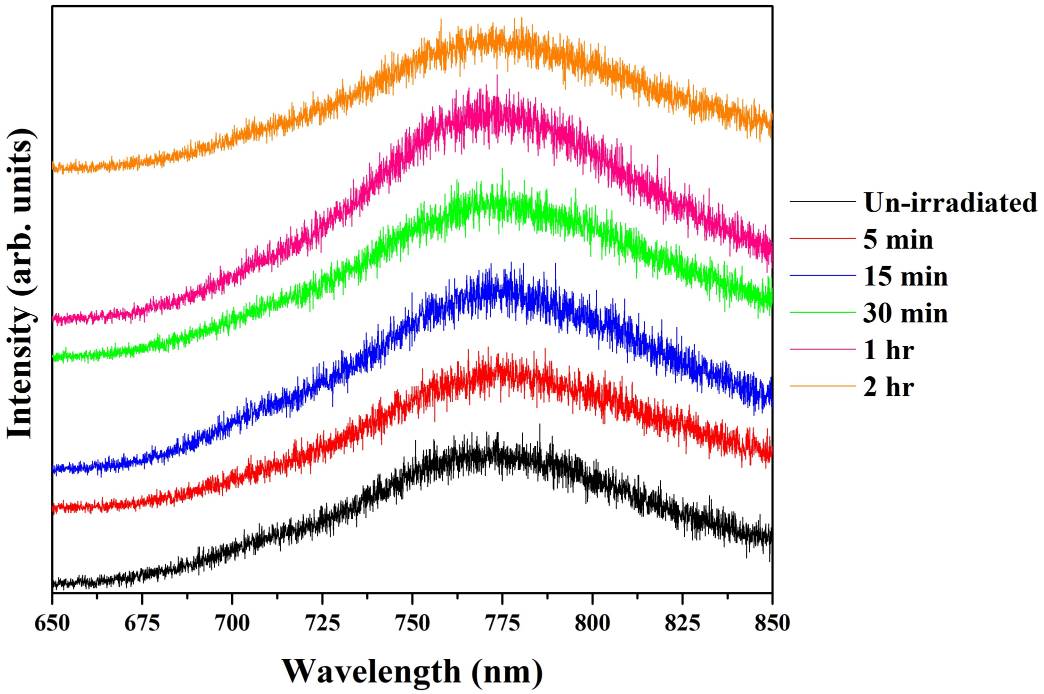

Figure S6. PL spectra of S2 exposed with UV for different duration.
